# Cellular Membrane Accommodation to Thermal Oscillations in the Coral *Seriatopora caliendrum*

**DOI:** 10.1371/journal.pone.0105345

**Published:** 2014-08-20

**Authors:** Chuan-Ho Tang, Lee-Shing Fang, Tung-Yung Fan, Li-Hsueh Wang, Ching-Yu Lin, Shu-Hui Lee, Wei-Hsien Wang

**Affiliations:** 1 National Museum of Marine Biology and Aquarium, Pingtung, Taiwan; 2 Institute of Marine Biodiversity and Evolutionary Biology, National Dong Hwa University, Pingtung, Taiwan; 3 Department of Sport, Health, and Leisure, Cheng Shiu University, Kaohsiung, Taiwan; 4 Institute of Marine Biotechnology, National Dong Hwa University, Pingtung, Taiwan; 5 Institute of Environmental Health, National Taiwan University, Taipei City, Taiwan; 6 Central of General Education, National Kaohsiung Marine University, Kaohsiung, Taiwan; 7 Department of Marine Biotechnology and Resources and Asia-Pacific Ocean Research Center, National Sun Yat-sen University, Kaohsiung, Taiwan; Duke University Medical Center, United States of America

## Abstract

In the present study, the membrane lipid composition of corals from a region with tidally induced upwelling was investigated. The coral community is subject to strong temperature oscillations yet flourishes as a result of adaptation. Glycerophosphocholine profiling of the dominant pocilloporid coral, *Seriatopora caliendrum*, was performed using a validated method. The coral inhabiting the upwelling region shows a definite shift in the ratio of lipid molecular species, covering several subclasses. Mainly, the coral possesses a higher percentage of saturated, monounsaturated and polyunsaturated plasmanylcholines and a lower percentage of polyunsaturated phosphatidylcholines. Higher levels of lyso–plasmanylcholines containing saturated or monounsaturated fatty acid chains were also revealed in coral tissue at the distal portion of the branch. Based on the physicochemical properties of these lipids, we proposed mechanisms for handling cellular membrane perturbations, such as tension, induced by thermal oscillation to determine how coral cells are able to spontaneously maintain their physiological functions, in both molecular and physical terms. Interestingly, the biochemical and biophysical properties of these lipids also have beneficial effects on the resistance, maintenance, and growth of the corals. The results of this study suggest that lipid metabolic adjustment is a major factor in the adaption of *S. caliendrum* in upwelling regions.

## Introduction

Organisms tend to have a tolerance that reflects the ambient conditions of their habitat; this tolerance can be due to either acclimation-related phenotype alterations or genotype selection during the adaptive process. Corals have adapted to geographic differences under ambient conditions through genetic shifts over long periods of time and have demonstrated the ability to acclimate at both the physiological and molecular levels [1]–[2]. However, corals are threatened by the extreme temperatures caused by climate change. Therefore, the extent of coral reef bleaching is of great concern. Thermal shock experiments have provided a better understanding of the physiological responses in coral by demonstrating how heating and chilling cause a breakdown of symbiosis. These temperature extremes induce the expression of stress proteins, retard energy transformation in the photochemical process, increase oxidative stress, and inhibit the photosynthetic activities of the zooxanthellae, leading to the elimination or departure of the zooxanthellae from the coral tissues in a process known as bleaching [3]–[4]. Therefore, processes such as coral growth and reproduction are greatly reduced by small thermal variations and even cause mortal dysfunction in serious situations [5].

Biological membranes play a major role in the function of a cell by forming a physical barrier, regulating and mediating the trans-membrane movement of specific ions and substances, and providing a matrix for multi-component assembly in metabolic and signaling pathways [6]. Membrane lipids constitute the basic structural elements for membrane function; these lipids polymorphically manipulate themselves to suit multiple requirements by displaying complex molecular interactions and physical properties. The dynamic state of the lipid molecules in a membrane is sensitive to ambient fluctuations such as thermally induced alterations in membrane lipid organization. These alterations present a serious challenge to the maintenance of physiological functions in organisms [7]–[8]. Nonetheless, membrane lipids have evolved to adopt many diverse molecular structures to form biological membranes with physical properties appropriate to the ambient circumstances and to restore membrane function following environmental insult. Membrane lipids achieve their molecular diversity by varying the arrangement of the chemical bonds within the structure. Indeed, alterations in lipid molecular structure can generate a membrane with entirely different properties through physicochemical mechanisms that exert a potent effect on biological processes [6], [9]–[11]. Thus, thermally induced perturbations in corals should result in alterations in the properties of biological membranes. Heat tolerance in corals has been correlated with a low ratio of polyunsaturated lipids in the membrane that maintain membrane integrity under heat stress. In contrast, a disrupted membrane composed of a high ratio of polyunsaturated lipids is found in heat-sensitive corals [12]. Similarly, coral bleaching and mortality caused by cold stress can also be related to the chill-induced loss of membrane integrity and the physiological dysfunction of biological membranes [13]. Therefore, the thermal history has potentially strong effects on the thermal susceptibility of corals [2].

Alterations in the order and phase state of membrane lipids are regarded as immediate variations in biological membranes during shifts in the ambient temperature [8]. Lipid molecules change their volumetric shape as they undergo thermally induced conformational order– or disorder–related changes; thus, a change in the thickness and area of a lipid lamella varies with the ambient temperature in the liquid–crystalline phase. Once a gel phase encounters a temperature below the main phase transition, lipid lamellae change drastically, resulting in lateral contraction. Therefore, a giant lecithin vesicle displays considerable surface area variance in a thermal–shift process, which implies leakage of the inner matter incident to the volume reduction of intracellular vesicles during the cooling process [14]. For vesicles of fixed volume and area, membrane tension increases in proportionally to the drop in temperature below the critical value where the membrane is unstressed, causing vesicle lysis at a reduction of ∼10°C [15]. Additionally, cooling–induced lipid phase separation leads to many interfacial regions; thus, the probability of pore formation with leakage should be greatly increased in the membrane [16]. During the warming process, the membrane mechanically relaxes by expanding its surface area; therefore, ensuing membrane distortions should be observable [14]–[15]. The current understanding is that membrane proteins should be suited to the membrane properties, such as tension, that are appropriate for initiating or optimizing membrane functions [9]. Consequentially, the properties of biological membranes are mechanically altered during temperature shifts and perturbations to the cellular homeostasis are expected.

Organisms that acclimatize to changes in their ambient conditions successfully exploit the diversity of the lipid molecular structure to meet the physiological requirements of specific circumstances. There is a metabolic cost to preserving or restoring membrane functions, and the inhabitants of a habitat with severe oscillations pay a large energy cost to maintain their physiological functions. However, coral communities with a high abundance of vital and fecund colonies flourish in tidally induced upwelling regions in Taiwan, where the seawater temperature varies sharply within a one-hour period [17]–[18]. It would be interesting, therefore, to determine whether the mechanism of adaptation of these corals can be elucidated. In this study, the membrane lipids in *Seriatopora caliendrum*, a dominant pocilloporid coral that inhabits areas near an upwelling region, were characterized. Alterations in lipid composition were related to the regulation of membrane functions and the physiological requirements for the coral to handle thermal oscillations. Selected lipid species critical for coral resistance, preservation, and growth are discussed further. The wound–healing rate was also measured to determine the vitality of the coral.

## Materials and Methods

### Field survey

**Figure 1:**
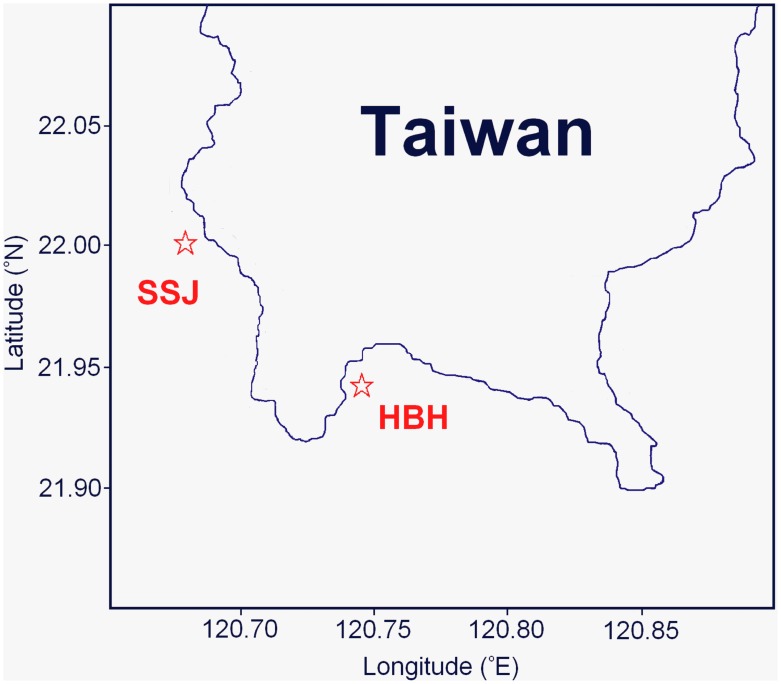
Location indications of the two study regions. The location of the upwelling (Houbibu, HBH) and the control (Siashuiku, SSJ) regions from which the coral samples were collected.

As shown in Figure 1, coral samples were collected from a tidally induced upwelling region (Houbibu [21°56′N, 120°45′E], HBH) and a control region (Siashuiku [22°1′N, 120°41′E], SSJ) in Kenting National Park, Taiwan in 2011 [17]. In the study period, 3–8 adult colonies of the pocilloporid coral *S. caliendrum* were collected from both study regions at a depth of 8–10 m. The collected coral colonies were immediately transported to the laboratory of the National Museum of Marine Biology and Aquarium near the study locations to measure the larvae yield [19]. The temperature variation of the ambient seawater was continuously recorded every 10 min *in situ* using a Hobo Water Temperature Pro logger (Onset Computer Corp., Bourne, MA, USA) during the study period. Using the magnitude of temperature oscillations and the comparability of the coral reproductive conditions of both study regions (see Results), coral samples collected during July were challenged to measure their significant adaptive responses to a fluctuating environment. A long–term environmental research project was launched by the National Museum of Marine Biology and Aquarium to regularly monitor the environmental health state of the coral reefs of the Kenting National Park. The recorded parameters of seawater quality provide data for further characterizing the variation in environmental conditions in the study regions [20]. The study and coral sampling were conducted under a permit issued by Kenting National Park Headquarters.

### Clade identification of zooxanthellates

Zooxanthellates DNA was extracted from the coral fragments, and the clades were successively identified according to the internal transcribed spacer rDNA sequence [21]. After the pre–treatment procedure, selected clones were sequenced and the obtained sequences were searched for and aligned in the NCBI GenBank Database with BLAST (http://www.ncbi.nlm.nih.gov/). High similarity between the cloned sequences of the zooxanthellates was found in the corals from the two study regions; these results (accession: KC684906 and KC684907) indicated that the zooxanthellates belong to clade C.

### Coral wound–healing experiment

Coral branches of equal size (∼2.5 g) were severed from the colonies derived from HBH and SSJ to concomitantly make a wound area of approximately 4 mm in diameter. Each coral branch was incubated in a 7–kl flow–through aquarium with shaded ambient light (<600 µE s^−1^ m^−2^). The temperature was maintained at 27±0.5°C by a thermal regulation circulatory system. The wound status was photographed during the experiment to compare the healing rate between the corals derived from each study region.

### Lipids analysis

After collection, a branch was obtained from each sample colony. The tip (0–0.5 cm from the branch end) and stalk (1–2 cm from the branch end) were collected, immediately frozen in liquid nitrogen and stored at −80°C. For pre–treatment and lipid extraction, the frozen coral fractions were ground in a liquid nitrogen–cooled mortar and then lyophilized overnight. The homogenous dry tissue was weighed and then extracted with a chloroform/methanol (2/1) solution. The mixture was then mixed thoroughly with a 0.15 M NaCl aqueous solution and the complete lower layer was collected for further analysis [22]. The coral storage lipids, triacylglycerol and wax ester, were analyzed using a method of high–performance thin–layer chromatography [23]. A validated method of reverse–phase high–performance liquid chromatography–electrospray ionization–triple quadrupole mass spectrometry was adopted for profiling the glycerophosphocholine (GPC) molecular species of the lipid extracts [22]. Briefly, the preliminary profiling of GPC was performed using a tandem mass spectrometry experiment, adopting a precursor ion scan of *m/z* 184 in the positive ion mode. Based on this phosphorylcholine–containing lipid profile, the product ion spectrum of each GPC species in the profile was acquired at their respective elution times. The molecular structure of the GPC in the sample was then determined by illustrating the product ion spectra of the [M+H]^+^, [M+Na]^+^ and [M+Ac]^–^ ions.

### Data analysis

The acquired GPC profile data were processed using the instrument software (Analyst 1.4; Applied Biosystems Instrument Co., USA) on the mass spectrometer. For peak detection, each molecular mass ion signal was manually determined from a total ion chromatogram of pooled coral samples during a time window of 0.2 min per step to extract the ion chromatogram. The extracted peak was recorded as *m/z* and retention time. After removing duplicated peak records, the remaining peaks were checked again to remove latent isotope peaks. A quantitation method was built using the final peak list to quantitate a batch of samples according to the Analyst 1.4 regulation procedure. Once a “Results Table” was obtained, all of the peaks in the batch of data were manually reviewed to confirm the accuracy of the peak alignment and peak area integration. The peak list, including the peak area of the extracted ion chromatogram, was finally exported as a Microsoft Excel file for further analysis.

After data processing, each detected signal was normalized to the total signal response in the GPC molecular species profile. Principal components analysis (PCA) using the SPSS 16.0 statistical software package (SPSS Inc., Chicago, Illinois, USA) was performed to determine whether metabolic differences between the GPC profiles in the corals could be distinguished. This pattern recognition method condensed the original set of variables to fewer variables based on their weighted averages; the new variables were termed scores and the weighted profiles were termed loadings. Each GPC profile was represented as a data point in the score plot and each point that was clustered together had a similar profile. The loading plot revealed information about the GPC molecular species that contributed to the data point separation in the score plot. A one-way analysis of variance (ANOVA) was used to further assess significant differences (*p*<0.05) of the sifted GPC molecular species in the loading plot.

### Lipid nomenclature

**Figure 2:**
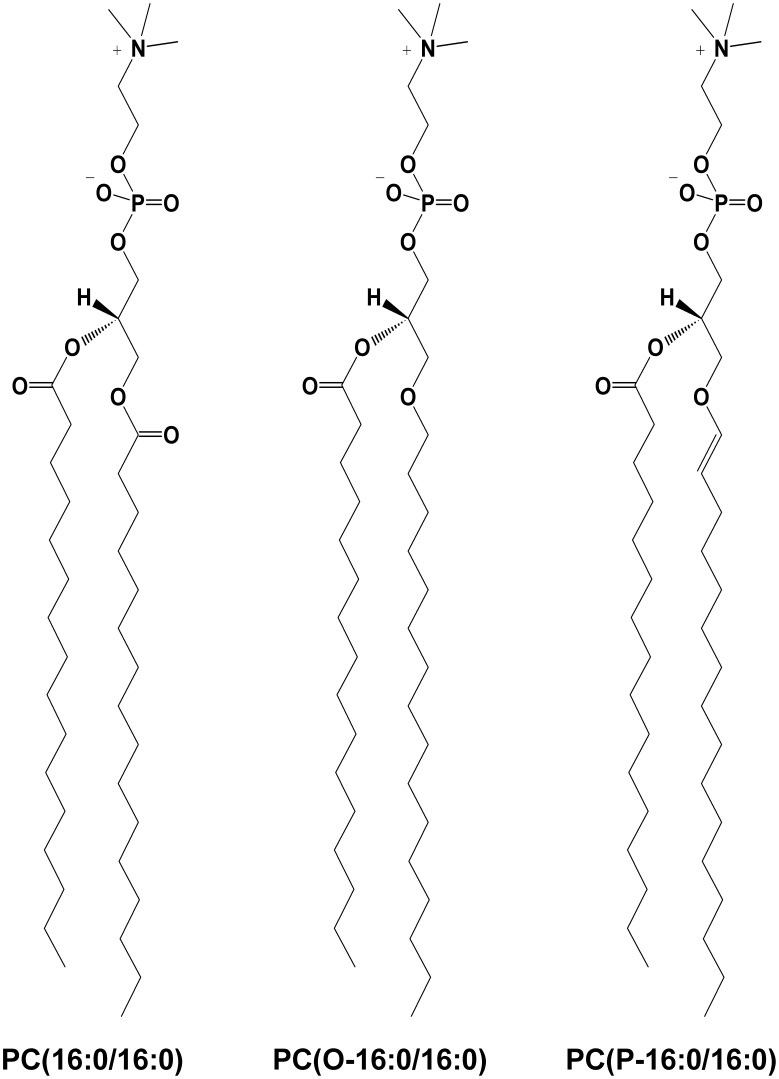
Structure of three glycerophosphocholine subclasses. PC(16:0/16:0), PC(O–16:0/16:0), and PC(P–16:0/16:0) are, respectively, indicated for 1,2–dihexadecanoyl–, 1–hexadecyl–2–hexadecanoyl–, and 1–(1Z–hexadecenyl)–2–hexadecanoyl– *sn*–glycero–3–phosphocholine.

Glycerophosphocholines are composed of a glycerol backbone with a phosphorylcholine head group at the *sn*–3 position and a fatty acid substituent at the *sn*–1 and *sn*–2 positions. The fatty acid substituent can be linked through an ester at both positions, and long–chain alkyl ether or vinyl ether linkages are also adopted at the *sn*–1 position. Because of the difference in linkages at the *sn*–1 position, the GPCs are further divided into the phosphatidylcholine (1,2–diacyl–GPC), plasmanylcholine (1–O–alkyl–2–acyl–GPC), and plasmenylcholine (1–O–alk–1′–enyl–2–acyl–GPC) subclasses. The designations and abbreviations of the GPC molecular species are based on the recommendations of the Lipid Metabolites and Pathways Strategy (LIPID MAPS). For example, 1,2–dihexadecanoyl–, 1–hexadecyl–2–hexadecanoyl–, and 1–(1Z–hexadecenyl)–2–hexadecanoyl– *sn*–glycero–3–phosphocholine were respectively abbreviated as PC(16:0/16:0), PC(O–16:0/16:0), and PC(P–16:0/16:0); molecular structures for these lipid are shown in Figure 2. The notation x:y for a fatty acid chain indicates the number of carbon atoms (x) and double bonds (y). In addition, symbols marked with ‘O’ or ‘P’ indicate a fatty acid–chain linkage with the glycerol backbone via the alkyl ether or vinyl ether, respectively, typically at the *sn*–1 position. In contrast, no marks indicate an ester linkage. In cases where both fatty acid chains cannot be assigned, the sum of their carbon atom and double bond numbers can be derived from deductive inference of the molecule once the lipid subclass was identified. These lipids were correspondingly abbreviated as PC(32:0), PC(O–32:0), and PC(P–32:0). The GPC molecular species that possessed a hydroxyl substituent at either the *sn*–1 or *sn*–2 position of the glycerol backbone were named lyso–GPC (LPC) (e.g., 1–hexadecanoyl–2–hydroxyl–*sn*–glycero–3–phosphocholines could be abbreviated as PC(16:0/0:0)).

## Results

### Temporal variations in seawater conditions

**Figure 3:**
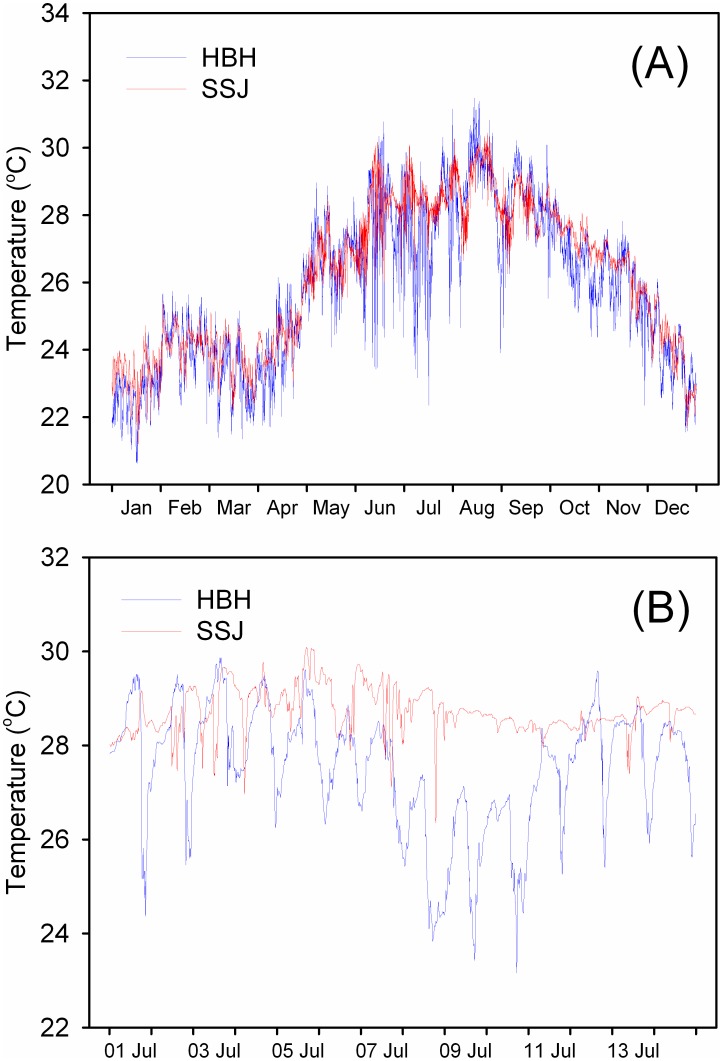
Temperature variation of seawater in the two study regions. Record of seawater temperature measured every 10 min in the upwelling (Houbibu, HBH) and control (Siashuiku, SSJ) regions inhabited by the corals from January–December 2011 (A). Subset of the temperature record between July 1 and July 14 (B) shows the temperature fluctuation in HBH.

The annual seawater temperature (Figure 3A) ranged from lower than 21°C to higher than 31°C. A sudden periodic drop in the temperature of the surface seawater was recorded in the coral reef located in HBH (the upwelling region). During the warm season, the temperature oscillated by as much as 6°C per day. The detailed oscillation in daily temperatures is shown in Figure 3B (a subset of the temperature record from between July 1 and July 14, 2011). Little daily oscillation of the seawater temperature was observed in SSJ (the control region).

The temporal variation in salinity, pH, turbidity, chlorophyll *a* content, five-day biochemical oxygen demand and nutrient content (NH_3_–N, NO_2_^−^–N, NO_3_^−^–N, PO_4_^3−^–P and SiO_2_–Si) in the ambient seawater of the coral habitats during the period of the present study are shown in Figure S1. As the long-term results show [20], the chlorophyll *a* and NO_3_^−^–N content in the seawater clearly differ between the two regions, with frequently higher values observed at HBH compared with SSJ. Notwithstanding, higher salinity with lower pH is generally also a feature of upwelling seawater, but these values varied to a similar extent in the two regions during the study. This observation can be explained by detailed characterization showing that such signals of upwelling seawater tend to be minimized near the coast [24].

### Seasonality in coral reproduction

**Figure 4:**
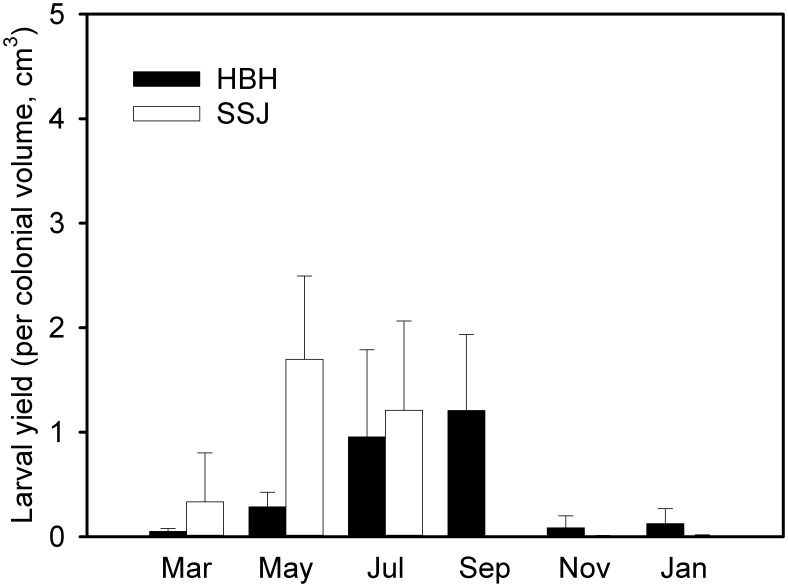
Seasonality of coral reproduction. Temporal variation of the larval yield of the coral colonies (n = 3–8, *Seriatopora caliendrum*) collected from the upwelling (Houbibu, HBH) and control (Siashuiku, SSJ) regions during Mar. 2011–Jan. 2012.

The yield of planula released from the coral colonies per month is presented in Figure 4. The coral reproduction shows marked seasonality, with higher larval yield during the summer and fall. Nevertheless, the timing of the peak yield for the HBH corals is later than that of the SSJ corals; a comparable larval yield was obtained during a partially overlapping period in July. The seawater temperature is considered to be a major factor in determining seasonal and spatial variations in coral reproduction [25]. A low seawater temperature may retard coral reproduction by increasing the planula development time. Tidal–induced upwelling brings cooler seawater from the subsurface layer to the surface, which may explain the reproductive delay observed in the HBH coral specimens.

### Storage lipid content

Similar levels of wax ester content in the corals (n = 3) inhabiting the HBH (1.48±0.01 µg/µg protein) and SSJ (1.43±0.11 µg/µg protein) regions were revealed (ANOVA, *p*>0.05). However, the triacylglycerol content in the corals from HBH (1.52±0.08 µg/µg protein) was approximately two–fold higher than that from SSJ (0.72±0.13 µg/µg protein; ANOVA, *p*<0.01).

### Glycerophosphocholine profile

**Figure 5:**
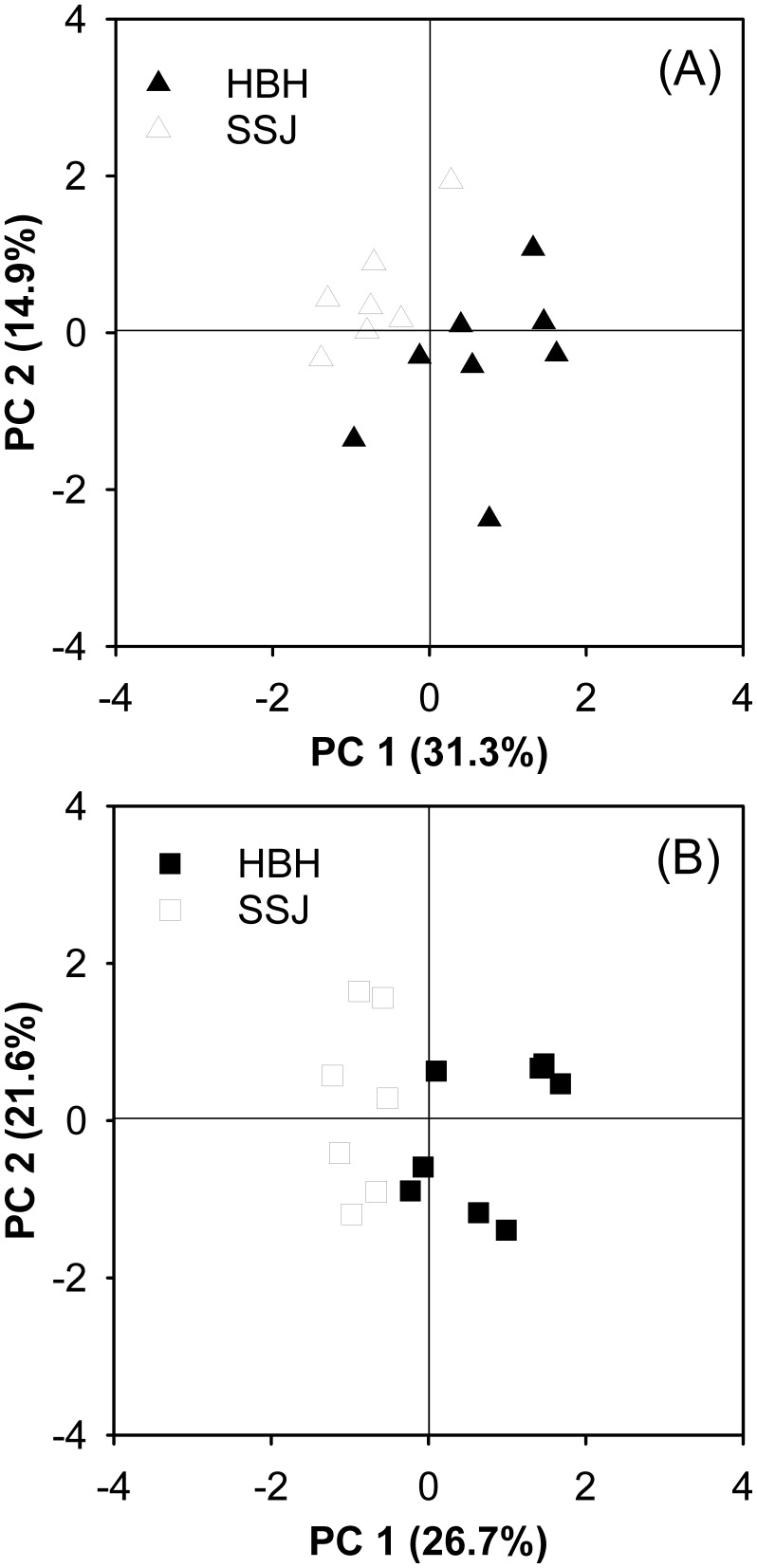
Visualization of the lipid profile differences in the corals. The score plot of principal component analysis, which showed distinguishable glycerophosphocholine profiles between the corals (*Seriatopora caliendrum*) inhabiting the upwelling (Houbibu, HBH) and control (Siashuiku, SSJ) regions in the tip (A) and stalk (B) portions of the tissue.

The profile of phosphorylcholine–containing lipids (see Table S1) was characterized through a three–replicate chemical measurement for each coral sample, and the difference in the lipid profiles between the corals was visualized as a PCA score plot. As shown in Figure 5, the swarms of data points belonging to the different groups are clearly divided, with the difference in the lipid profiles clearly distinguishing between the corals inhabiting the HBH and SSJ regions. Both the first and second principal components contributed to the group separation in Figure 5A, which described a variation of 31.3% and 14.9%, respectively, in the dataset derived from the tip position of the coral tissues. With regard to the stalk position, as shown in Figure 5B, the first principal component described a variation of 26.7% in the dataset contributing to the group separation. Each correlation loading plot revealing important GPC species is shown in Figure S2 and S3, respectively.

**Figure 6:**
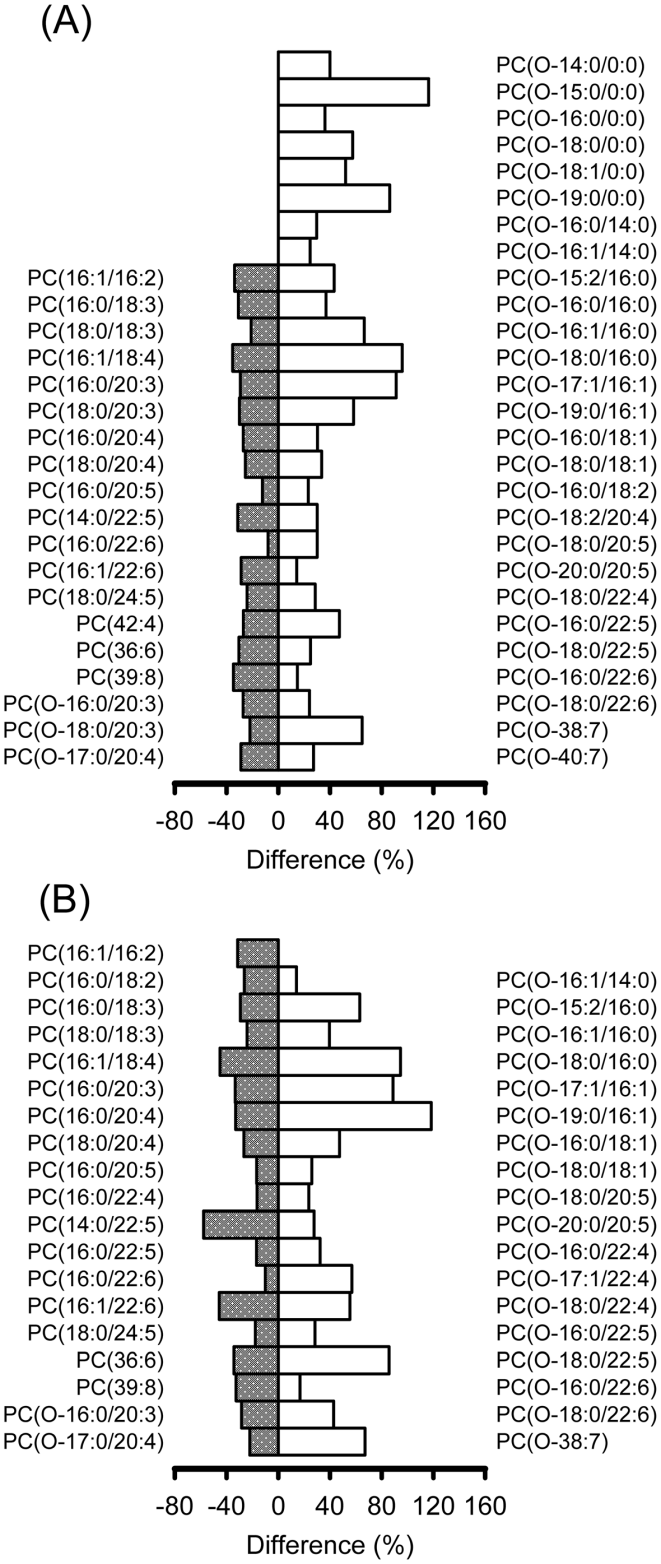
Selected lipid species relative to the regulation of thermal–oscillatory acclimation by the coral. The difference in glycerophosphocholine proportions (ANOVA, *p*<0.05) in the coral *Seriatopora caliendrum* sampled from the upwelling region (Houbibu) compared with the coral specimens from the control (Siashuiku) region in the tip (A) and stalk (B) portions of the tissue.

More than 150 GPC molecular species were identified in the lipid profile of the corals (see Table S1). As shown in Figure 6, the coral from HBH had a significantly higher proportion of plasmanylcholines (ANOVA, *p*<0.05) that are saturated (29.7–95.9% higher) or included a low (1–2 double bonds, 14.1–118% higher) or high (4–6 double bonds, 14.2–85.7% higher) degree of unsaturated fatty acid chains. Nevertheless, the proportion of polyunsaturated phosphatidylcholines in the coral derived from HBH was 7.9–57.8% less than that derived from SSJ (ANOVA, *p*<0.05). Some plasmanylcholine species possessing 20–carbon chains with 3 or 4 double bonds were also found in a lower proportion (21.8–28.9% lower; ANOVA, *p*<0.05) in the coral from HBH. Additionally, the coral from HBH contained a higher proportion of lyso–plasmanylcholines (36.1–116% higher; ANOVA, *p*<0.05) in the tip position tissue. Although some lyso–plasmanylcholines in the stalk position of the corals showed a notable weight in the PCA grouping (see Figure S3), the difference was not significant according to the ANOVA analysis (*p*>0.05).

### Coral wound healing

**Figure 7:**
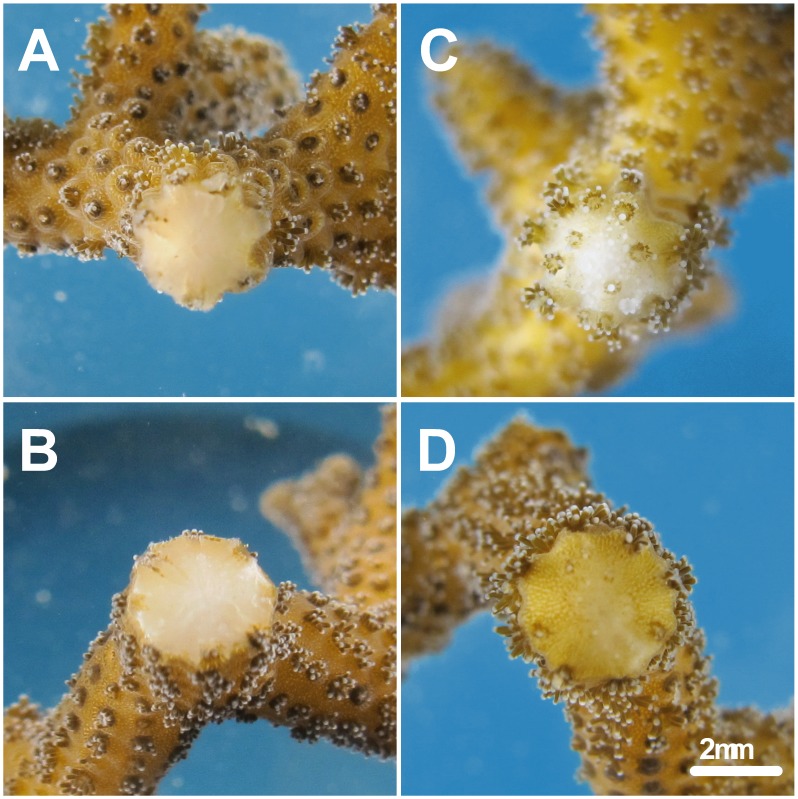
Difference in wound–healing rates in the corals inhabiting the two study regions. Wound images in the coral fragments from the *Seriatopora caliendrum* specimens sampled from Houbibu (A, C) and Siashuiku (B, D) on day 1 (A, B) and day 15 (C, D) of the healing experiment.

A three–replicate experiment was performed to compare the wound–healing rate of the corals derived from each study region. As shown in Figure 7 A and B, a wound approximately 4 mm in diameter was created when the coral branches were collected from the colony. In the coral regeneration process, the expansion of tissue from the boundaries of the wound is correlated with time. The time required by the coral tissue for full recovery of the wound area was recorded for estimating the wound–healing rate. As shown in Figure 7 D, healing was defined as the point when a thin coenosarc covered the wound in the coral branch and polyps were initially developing. Based on this definition, the coral derived from HBH (6.0±1.0 days) exhibited a considerably faster wound–healing rate than that of the coral derived from SSJ (15.7±0.6 days; ANOVA, *p*<0.001). Images of the coral wound status revealed a sharp contrast in the healing between the corals from the two regions. As shown in Figure 7 C, 15 days after the challenge, the wounded site of the coral branch derived from HBH developed packed polyps and an expanded skeleton that formed a completely new growing point.

## Discussion

The coral community near HBH experiences cyclical temperature variations of up to 6°C with a shifting rate of approximately 3–9°C/h as a distinguishing environmental feature. The HBH coral must manage unfavorable effects arising from the cooling–induced phase separation in the membrane, such as membrane thickness mismatch at the phase boundary and lateral contraction–induced tautness [26]–[27]. The higher proportion of plasmanylcholines and lyso–plasmanylcholines in the HBH coral can be correlated with the regulation induced by temperature oscillation. Although a decrease in the amount of phosphatidylcholines with polyunsaturated chains containing fewer than five double bonds is expected, an increase in polyunsaturated plasmanylcholines with more unsaturated and/or prolonged chains was observed because such chains have an intrinsic propensity toward a high degree of conformational flexibility for structural packing to simultaneously regulate the surface area and thickness of the membranes [28]. Through their conformational flexibility, polyunsaturated chains endow the membrane with a greater capacity to match the phase boundary between the lipid domains and thus counterbalance the membrane tension that accompanies thermal shifts. Moreover, based on geometrical packing LPC can result in a spontaneous tendency toward a monolayer with positive curvature, thus smoothing and stabilizing the phase boundary to avoid further hydrophobic region exposure [26], [29]. An increase in the number of plasmanylcholines with a low degree of unsaturated chains can enhance the mechanical strength and stability of the membrane to resist tension–induced leakage [30]–[32]. It remains to be confirmed whether the self–assembly of plasmanylcholines from a bilayer structure into a fully interdigitated arrangement is compatible within a living cellular membrane and can thereby counterbalance the thermal shift–induced tension perturbation [33].

During cooling, polyunsaturated lipids should be concentrated in the requisite regions of the membrane, which dramatically enhances the permeability [31], [34]–[35]. Replacing the carbonyl group with methylene allows plasmanylcholines to provide the membrane with an enhanced barrier against ion and water permeation [36]–[37]. This replacement also augments the double bond–induced shift in lateral membrane pressure [38]. Thus, corals may offset the cooling–induced opposite shift in lateral pressure but avoid excess membrane fluidity by raising the unsaturated levels when the ambient temperature shifts back to the normal surface water level [39]. Accordingly, the resistance of the HBH corals may be elevated from the thermally induced increase in oxidative stress and intracellular calcium perturbations [4]; as a result, the corals appear to be less sensitive to temperature variations and are largely unaffected in terms of physiological responses across a broad range of thermal treatments [40]–[41].

Calcification in scleractinian corals requires the transport of many materials, including calcium, inorganic carbon, proton, and an “organic matrix” [42]. Lyso–GPCs have been reported to be good activators of calcium-ATPase and L-type ion channels through their direct actions altering membrane characteristics to stimulate calcium ion flux activity [43]–[44]. Due to their inverted cone molecular shape, LPCs have been regarded as important elements in membrane vesiculation and fusion and are, therefore, able to facilitate vesicle–mediated transportation in the secretion of “organic matrix” [45]–[46]. Coral growth involves both cell growth and skeleton formation, and plasmanylcholine levels have been reported to positively correlate with accelerated cell growth and the level of cellular differentiation [47]–[48]. Furthermore, the depletion of polyunsaturated lipids accompanied by the promotion of monounsaturated lipids and LPC production resulted in accelerated cell growth [49] in the HBH coral specimens compared to the SSJ specimens. This result was clearly revealed by the rapid repair of injury by the HBH coral (Figure 7). In the upwelling region, therefore, the prospering coral community was correlated with an increase in the beneficial lipid mixtures, favoring calcification and cell growth. In addition, a sufficient reserve of triacylglycerol can be, or can result from, an adaptive regulation in the HBH corals to address the recurrent variation in ambient temperature. For instance, the alteration in the composition of the membrane lipids in the HBH corals allows more efficient cell maintenance and functioning and reduces energy costs. Thus, these corals can maintain a greater reserve of triacylglycerol for faster growth and better reproduction [18].

In summary, the alteration in lipid composition was related to the maintenance of membrane functions and to the physiological requirements for the corals inhabiting the upwelling region to handle thermal oscillation–induced cellular membrane perturbations. As a possible mechanism for environmental acclimation, the cellular membrane's accommodation of strong cyclical temperature variations is described in both molecular and physical terms. Additionally, the corals have acclimatized to the thermal oscillation and achieved competitiveness, now thriving in the upwelling region. This result provides a new way of thinking about coral community development that can be related to the improvement of cellular fitness through the acclimation regulation of membrane lipid metabolism. However, further studies are required to incorporate more complete profiling of lipid classes or measurements of membrane bulk properties to gain insight into the mechanism by which the cellular membrane accommodates thermal oscillations.

## Supporting Information

Figure S1 **The temporal variation of seawater quality.**
(DOC)

Figure S2 **The correlation loading plots of latent variables 1 and 2, generated from the PCA model, which show lipid variations in the tip position of the corals (** ***Seriatopora caliendrum*** **) contributing to the data point separation in the score plot.**
(DOC)

Figure S3 **The correlation loading plot of latent variable 1 generated from the PCA model, which shows lipid variations in the stalk position of the corals (** ***Seriatopora caliendrum*** **) contributing to the data point separation in the score plot.**
(DOC)

Table S1 **The percentage of glycerophosphocholine species detected in the coral (** ***Seriatopora caliendrum*** **).**
(XLS)
