# Supplementary material for: Cellular Membrane Accommodation to Thermal Oscillations in the Coral Seriatopora caliendrum
Source: PLoS One. 2014 Aug 20;9(8):e105345. doi: 10.1371/journal.pone.0105345 (PMC4139334; doi:10.1371/journal.pone.0105345)
Supplement: Figure S1 — The temporal variation of seawater quality. (DOC) [file pone.0105345.s001.doc]

**Figure S1.**  **The temporal variation of seawater quality.** Variation in salinity, pH, turbidity, chlorophyll *a* (chl. *a*), five-day biochemical oxygen demand (BOD_5_) and nutrients (NH_3_–N, NO_2_^–^–N, NO_3_^–^–N, PO_4_^3–^–P and SiO_2_–Si) in the seawater of the upwelling (Houbibu, HBH) and control (Siashuiku, SSJ) regions during 2011.
