# Supplementary material for: Cellular Membrane Accommodation to Thermal Oscillations in the Coral Seriatopora caliendrum
Source: PLoS One. 2014 Aug 20;9(8):e105345. doi: 10.1371/journal.pone.0105345 (PMC4139334; doi:10.1371/journal.pone.0105345)
Supplement: Figure S3 — The correlation loading plot of latent variable 1 generated from the PCA model, which shows lipid variations in the stalk position of the corals ( Seriatopora caliendrum ) contributing to the data point separation in the score plot. (DOC) [file pone.0105345.s003.doc]

**Figure S3.** The correlation loading plot of latent variable 1 that was generated from the PCA model, which show lipid variations in the stalk position of the corals (*Seriatopora caliendrum*) contributing to the data point separation in the score plot. The peak signals which molecular species had not been identified are indicated as “*m/z*@retention time”.
